# A Paediatric Perspective: Opportunities and Challenges in Emergency Department Antimicrobial Stewardship

**DOI:** 10.3390/antibiotics15010071

**Published:** 2026-01-09

**Authors:** Karen N. McCarthy, Kara Tedford, Eimear Kitt

**Affiliations:** 1 Department of Infectious Diseases, Children’s Health Ireland (CHI), D12 N512 Dublin, Ireland; karen.nmccarthy@childrenshealthireland.ie; 2 Department of Pharmacy, Children’s Health Ireland (CHI), D12 N512 Dublin, Ireland; kara.tedford@childrenshealthireland.ie; 3 School of Pharmacy and Biomolecular Sciences, Royal College of Surgeons Ireland, D02 YN77 Dublin, Ireland; 4 Department of General Paediatric, Children’s Health Ireland (CHI), D12 N512 Dublin, Ireland

**Keywords:** antimicrobial stewardship, paediatric, emergency department

## Abstract

The Emergency Department (ED) represents an ideal location for antimicrobial stewardship (AMS) intervention, given the large volume of antibiotics seen prescribed to a wide variety of patients. This is particularly true in paediatrics, where most infectious presentations are viral in nature. A recent European Society of Clinical Microbiology and Infectious Diseases (ESCMID) position paper addressed four key areas affecting adult ED. This included: (1) the utility of biomarkers or rapid pathogen tests, (2) the impact of blood cultures on antibiotic prescribing, (3) the effect of watchful waiting on clinical outcomes, and (4) the potential for structured follow-up programmes within the ED to impact prescribing. Comparatively, the paediatric ED remains underrepresented in the literature with regard to AMS interventions. In this review article, we review the evidence surrounding the above four key areas as they relate to the paediatric population.

## 1. Introduction

Antimicrobial stewardship (AMS) is an internationally recognised strategy that addresses antimicrobial resistance (AMR) by encouraging appropriate antibiotic use, thus maximising optimal clinical outcomes and minimising adverse events [1]. The Emergency Department (ED) represents an ideal location for AMS intervention given that it serves as the primary source of hospital admissions with a large burden of patients with potential infections [2]. This is particularly true in paediatrics where the ED sees a diverse population including many acute ambulatory encounters, with the majority of infectious presentations being viral in nature and not requiring antibiotic treatment. Antibiotics are the most commonly prescribed medicines in paediatrics. Almost 10% of children attending the emergency department receive a prescription for antibiotics, increasing to more than 30% if the presenting complaint is fever [3,4]. The efficient turnaround critical to the successful operation of a paediatric ED (PED) relies on focused decision making, to which evidence-based diagnostic capacity is key. However, many challenges exist unique to the PED that include diagnostic uncertainty, both clinical and biochemical, diversity of AMS expertise, inconsistent access to follow-up care and communication to primary providers, and perceived patient and parent expectations [4].

A recent ESCMID position paper addressed four key areas affecting adult EDs [5]. This included (1) the utility of biomarkers or rapid pathogen tests, (2) the impact of blood cultures on antibiotic prescribing, (3) the effect of watchful waiting on clinical outcomes, and (4) the potential for structured follow-up programmes within the ED to impact prescribing. The key recommendations of that paper concluded that we were to empower clinicians to reach a quick and accurate diagnostic using a combination of clinical expertise, rapid diagnostics including biomarkers, and imaging, but maintain an appropriate degree of diagnostic stewardship, that includes prompt and proper collection of blood cultures in optimal patient populations in advance of antibiotic therapy. Delayed prescriptions were encouraged when appropriate, and designated follow-up that is evidence/guideline-based should be performed by trained staff [5].

Comparatively, despite the PED initiating a significant proportion of the antibiotics prescribed for admitted patients with infection, it remains underrepresented in the literature with regard to AMS interventions. Specific guidance is challenging to implement, with most recommendations based on low certainty of evidence. The scarcity of high-quality AMS studies in PEDs highlights the urgent need for future research in this field. In this article, we will review the evidence and unique challenges posed to the paediatric population with regard to AMS in paediatric EDs.

## 2. Are Biomarkers Helpful in Reducing Unnecessary Antibiotic Prescribing in the Paediatric Emergency Department?

### 2.1. C Reactive Protein

C reactive protein (CRP) is an acute phase reactant that is commonly used in clinical practice to assess the probability of bacterial infection. It is a protein synthesised by the liver in response to inflammatory stimuli, both infectious and non-infectious. CRP levels rise within four to six hours of a stimulus, doubling every eight hours and peaking at 36 to 50 h. In general, values of less than 5 mg/L suggest a low risk of serious infection; however, no single CRP cut-off value for serious bacterial infection in paediatric populations has been defined, despite widespread clinical use. CRP testing has several limitations; in particular, the kinetics, with a delayed rise and fall in levels with onset and resolution of illness, and a lack of specificity and inter-individual variability. CRP values greater than 50 mg/L are independently associated with a higher rate of antibiotic prescription on discharge from the PED [6]; however, studies to evaluate the utility of CRP in stewardship interventions in the PED are lacking. A stepped-wedge randomised controlled trial evaluated CRP as well as clinical characteristics as part of a clinical prediction model aiming to reduce antimicrobial prescriptions for lower respiratory tract infection (LRTI) in children presenting to a PED. The intervention did not reduce overall antibiotic prescription; however, a reduction in antibiotic use in low-/intermediate-risk children and a reduction in treatment failure in high-risk children may reflect more appropriate prescribing with this CRP-guided model [7]. A Cochrane review, in a primary care setting, concluded that point-of-care CRP may reduce antibiotic use for acute respiratory traction infections, but the degree of the effect remains unclear [8]. The recent ESCMID position paper recommended against the use of CRP in the ED to guide the initiation of antibiotics for patients with respiratory tract infection, citing a lack of evidence. At present, the paediatric perspective is similar, with a lack of data to support CRP-guided antibiotic decision making in the PED.

### 2.2. Procalcitonin

Procalcitonin (PCT) can be induced during infection in response to stimulation by pro-inflammatory cytokines and bacterial endotoxins [9,10]. Notably, interferon-γ, produced prominently during viral infection, downregulates IL-1-β-induced PCT production, which is why higher levels are typically observed during bacterial rather than viral infections [11]. Furthermore, PCT kinetics are diagnostically favourable as levels increase faster following the onset of bacterial infection (4–6 h) and peak earlier (12–24 h) when compared to CRP.

There is increasing evidence for the utility of PCT as an AMS tool in adult patients. The ADAPT-sepsis randomised clinical trial recently demonstrated that a daily PCT-guided protocol reduced total antibiotic duration compared to the standard of care among hospitalised critically ill adults with suspected sepsis [12]. A Cochrane review summarising 26 RCTs of PCT use in adult community acquired pneumonia (CAP) concluded that its use is associated with 15% less antibiotic initiation, reduced treatment duration, and lower antibiotic associated adverse effects [13]. Indeed, as summarised in the recent ESCMID position paper, the use of PCT to predict disease severity and guide initiation of antibiotics is recommended among adult patients with CAP, asthma, and exacerbations of COPD who are likely to be admitted to hospital [5].

So, what does this mean for paediatrics? In the post-vaccine era, the majority of paediatric CAP cases are caused by viruses; however, antibiotic use continues to account for a significant number of inpatient days among paediatric patients [14,15]. A biomarker to distinguish between bacterial and viral aetiologies would therefore have potentially greater AMS benefit among younger patients. However, the literature is not as convincing. Higher serum PCT values are associated with chest X-ray abnormalities and complications, e.g., pleural effusion; however, the cost–benefit of the routine addition of PCT to existing diagnostic criteria is unclear [16]. In a sub-analysis of the EPIC study, no child with a typical bacterial CAP had a PCT value of >0.1 ng/mL. Here, a threshold of >0.25 ng/mL was determined to be 85% sensitive and 45% specific for identifying children with bacterial CAP. While PCT values were higher among children with pneumonia admitted to ICU and with complicated CAP, 64% of children with only viral pathogens detected had a PCT value of >0.5 ng/mL, a threshold that defines a high likelihood of bacterial disease in an adult cohort [17]. In contrast to the adult data, PCT-guided algorithms have not convincingly demonstrated utility in reducing antibiotic prescribing in paediatric CAP in the ED to date. In a Swiss RCT recruiting 337 children, antibiotic initiation was not different among PCT-guided and control patients; however, the use of serial PCT was associated with a reduction in the duration of antibiotics in the PCT-guided group (4.5 vs. 6.3 days) [18]. In contrast, an Italian RCT demonstrated a 15% reduction in antibiotic initiation among paediatric CAP patients using a PCT cut-off value of 0.25 ng/mL [19].

In summary, in contrast to the more robust adult data, there is currently insufficient and conflicting evidence to support the use of PCT alone in the PED to guide antibiotic initiation or to define disease severity in CAP. Studies on the clinical utility of these biomarkers are hindered by the fact that diagnostic lower respiratory tract samples are difficult to obtain in children and clinical judgement remains the diagnostic gold standard. However, PCT may be useful as a decision aid among children with very low values (<0.1 ng/mL), and serial measurements among inpatients may help to guide antibiotic duration.

### 2.3. UTI

Urinary tract infection is a common presentation in paediatrics. Current point-of-care diagnostic strategies, including urinary dipstick analysis and microscopy, lack sensitivity and definitive diagnosis with culture results may delay appropriate antibiotic use. An important clinical differentiation in the paediatric ED is whether the patient has a localised infection with cystitis alone or a complicated urinary tract infection with acute pyelonephritis [20]. PCT has demonstrated a higher sensitivity and specificity than CRP and white cell count for differentiating acute pyelonephritis and lower UTI among paediatric patients with febrile urinary tract infection [21]. Procalcitonin also correlates with severity of renal involvement, likelihood of renal parenchymal injury, and clinical response, with a cut-off value of >1.0 ng/mL suggested as being associated with an increased risk of parenchymal injury [21,22,23]. A meta-analysis found that a PCT > 0.5 ng/mL yielded a 71% sensitivity and 72% specificity for acute pyelonephritis and was significantly associated with late scarring (79% sensitivity, 50% specificity) [24]. Therefore, while PCT measurement at admission may be helpful in predicting renal parenchymal injury in paediatric pyelonephritis and may stratify who needs additional renal imaging, there is currently insufficient evidence to support its use as an AMS tool in EDs.

### 2.4. Febrile Neonate

Infants under three months of age presenting with fever are at high risk of serious bacterial infection (SBI), such as sepsis, meningitis, or urinary tract infection, with an increased risk among those less than one month of age. Diagnostic evaluation of this group is invasive, requiring blood testing and often lumbar puncture, and this presents a significant burden on the paediatric ED workload, antibiotic use, and inpatient bed days. Multiple scoring systems and diagnostic algorithms exist to guide the management of the febrile infant; however, definitive identification of infants at low risk of SBI could potentially avert invasive investigations, antibiotic initiation, and hospital admission [25,26,27,28]. Furthermore, due to parental awareness about the significance of pyrexia in this group, the duration of illness prior to presentation is often short, for which the kinetics of PCT with its earlier production than other biomarkers of infection may be advantageous.

In a retrospective study, a serum PCT > 0.3 ng/mL had a sensitivity and specificity of 89.5% and 87.5% for predicting serious bacterial infection in infants under 90 days presenting to the ED [29]. PCT has been demonstrated to have either comparable or improved diagnostic accuracy when compared to CRP for the diagnosis of invasive bacterial infection; for example, bacteraemia and meningitis, in febrile infants [30,31]. Milcent et al. suggest that febrile infants less than one month of age, for whom under current guidelines, a lumbar puncture would be recommended, could avoid this intervention if the serum PCT was <0.3 ng/mL, suggesting a low risk of invasive bacterial infection [30]. Using a step-by-step approach, whereby clinical characteristics and laboratory tests in addition to PCT are used to risk stratify patients, the use of a PCT > 0.5 ng/mL, in addition to other clinical and laboratory measures (well-appearing, age > 21 days, absence of leukocyturia, CRP < 20 mg/L, and ANC < 10,000/mm^3^) reduced the misclassification of infants with serious bacterial infection as low risk from 1.2% to 0.5% [32].

A limiting factor in the implementation of PCT for risk stratification of febrile infants less than three months is that a definitive cut-off value has not been defined. In a large multi-centre European RCT, a PCT cut-off of 0.5 ng/mL was found to be the only independent risk factor for invasive bacterial infection (positive CSF or blood culture) among febrile infants less than three months old. Maniaci et al., however, suggest a cut-off of 0.12 ng/mL for identifying definite and possible serious bacterial infection in this age group [32]. Rotrosen et al. found that among infants < 60 days, serum PCT < 0.17 ng/mL, negative urinalysis, and ANC of <4090/μL had 97.7% sensitivity and 60% specificity for ruling out serious bacterial infection [33].

In summary, PCT likely enhances the accuracy of risk stratification of febrile infants less than three months of age, but the clinical context and other laboratory measurements remain an important part of the diagnostic algorithm in this high-risk cohort. Defining a consistent cut-off for PCT in this patient group is an important next step in the implementation of this evidence into broader clinical practice in the ED.

### 2.5. Other Clinical Scenarios

There is also evidence for the utility of PCT in other clinical scenarios in paediatrics. Conflicting evidence exists among paediatric patients with sickle cell disease (SCD) presenting with vaso-occlusive crisis (VOC) for PCT. A PCT < 0.5 ng/mL was found in one study to have a high negative predictive value for bacterial infection (100%) [34]. However, no association with invasive bacterial infection was described in two more recent studies, with the conclusion that PCT levels are often elevated in SCD patients experiencing VOC due to the associated systemic inflammatory response, and that this was not associated with bacterial infection in this cohort [35,36]. Among children with central venous catheters (CVC) presenting with fever, a PCT of >0.6 ng/mL was found to be 85.6% sensitive and 65.7% specific at predicting bacteraemia [37]. Another study, using a cut-off of 0.3 ng/mL, demonstrated a 93% sensitivity and 63% specificity in the same clinical scenario [38]. Therefore, PCT may have utility in the ED to triage febrile children with CVC in situ; however, further prospective studies are required.

### 2.6. Combination Testing

Given the lack of specificity of PCT alone in paediatric CAP, further studies have evaluated combinations of interventions to guide antibiotic decision making in EDs. An RCT, using a combined PCT, Flu/RSV assay, and pharmacist-led education, among patients attending ED for influenza-like illness or acute respiratory illness, found that these interventions did not affect antibiotic or antiviral prescriptions in the subset of paediatric patients studied [39]. Other combination scores such as the Labscore (PCT, CRP, and urine dipstick) have also been evaluated for performance in differentiating bacterial from viral infection in the paediatric ED. These combination biomarkers have demonstrated superior sensitivity and or specificity to any individual biomarker among paediatric patients less than three years old presenting with fever without a source [40].

### 2.7. Emerging Biomarkers

Another biomarker combination (ImmunoXpert), which measures tumour necrosis factor-related apoptosis-inducing ligand (TRAIL), interferon gamma induced protein-10 (IP-10), and C-reactive protein (CRP), demonstrated comparable AUC for predicting bacterial infection [41]. TRAIL, interferon-gamma and lipocalin 2 (LCN2), and IL-6 in combination also perform well in differentiating bacterial from viral infection among febrile children in the ED [41]. In paediatric CAP, other novel biomarkers have been identified to predict the severity of disease, such as angiopoietin-2 levels, soluble triggering receptor expressed on myeloid cells 1 (sTREM-1), and IL-8 [15,42,43]. Host gene expression signatures in blood also demonstrate promise in differentiating viral from bacterial infection in children. For example, using a machine learning approach in combination with whole blood transcriptomics, 161 transcripts can be used to classify febrile children to 18 infectious or inflammatory disease categories [44].

Presepsin is another promising biomarker. It is a soluble CD14 subtype that is a generated during pathogen recognition by innate immune cells. Given that its production is stimulated at the early stages of pathogen invasion, presepsin provides a potential advantage over other biomarkers with rises expected three hours post-infection. A recent meta-analysis exploring the utility of presepsin in paediatric cohorts found excellent pooled sensitivity of 0.87 (95% CI: 0.81–0.88), specificity of 0.86 (95% CI: 0.80–0.90), and AUC of 0.91 (95% CI: 0.88–0.93). Excellent diagnostic performance was noted among neonates, where other biomarkers are less robust [45]. However, significant variability was noted as a limiting factor in this meta-analysis, and indeed, another recent study exploring the clinical utility of presepsin for discriminating between bacterial and viral infections in young infants in PEDs noted limited discriminative value, albeit with a small sample size [46].

In conclusion, no single marker can replace clinical judgement in any situation, particularly in the paediatric population. Combination markers may perform better; however, more research is needed. Novel biomarkers are promising but are not yet routinely available. Finally, this evidence highlights the need for caution in applying adult data directly to paediatrics, as this approach could result in a net increase in antibiotic consumption.

## 3. Does Taking Blood Cultures in Common Infectious Syndromes Improve Antibiotic Prescribing and/or Clinical Outcomes?

Increasing evidence suggests that routinely obtaining blood cultures in the majority of well-appearing children with common infectious presentations is not recommended. Despite this, it is commonly performed in practice, often adding little value and introducing potential harm in the form of prolonging hospital stays from potential contaminants and clinically irrelevant positive cultures.

ESCMID supports not obtaining blood cultures in otherwise immunocompetent individuals with non-severe CAP, UTI, and skin and soft tissue infection (SSTI). These recommendations are supported by paediatric literature, including the PIDS/IDSA guidelines for pneumonia, where it is suggested to perform them in inpatients with moderate to severe pneumonia only [47], and the IDSA guidelines on SSTI management [48]. Similarly, blood cultures add little value in the management of UTIs in children over the age of three months who are otherwise well appearing [39,48,49,50,51,52]. For complicated UTIs, consideration may be given depending on the clinical scenario. The current ESCMID guideline suggests obtaining a blood culture in severe presentations when sepsis is a concern [5]. Indeed, bacteraemia is a known occurrence in up to 9% of infants with UTI. With the exception of possibly longer durations of febrile episodes, clinical symptoms do not significantly distinguish bacteraemic from non-bacteraemic UTI in children, and outcomes have been comparable [52].

However, in line with ESCMID, international guidelines agree that in patients with severe presentations, pyrexial infants aged less than 3 months, immunocompromised hosts where there is concern for unusual or resistant pathogens and in situations where there is either abnormal anatomy or indwelling prosthetic material, a blood culture is recommended as an important tool for diagnostic and therapeutic reasons. This is also true where the patient may have been partially treated with antibiotics or had failed to respond appropriately [47,48].

## 4. Is Watchful Waiting a Useful Approach to Reduce Antibiotic Use in Paediatric Patients?

Delayed prescribing or watchful waiting is a strategy to reduce antimicrobial use whereby the prescription or dispensing of an antibiotic is delayed, allowing for symptoms to improve spontaneously. This is beneficial as it can reduce unnecessary antibiotic use, antibiotic associated adverse effects, and healthcare costs [53]. For example, for acute respiratory tract infections likely to be caused by a virus, delayed prescriptions are not associated with any difference in clinical outcome or significant reduction in patient satisfaction when compared to immediate prescription or no prescription [54,55]. However, in this scenario, as no prescription is associated with similar clinical outcomes and patient satisfaction with a lower rate of antibiotic use, this is favourable and endorsed in many guidelines [56].

Acute otitis media accounts for a significant burden of outpatient paediatric antimicrobial prescriptions. Watchful waiting is now the initial strategy recommended for the management of uncomplicated acute otitis media (AOM) in many national and international guidelines [57]. However, in a systematic review, it was observed that adherence with this approach is poor, with antibiotics prescribed for between 44.8% and 98% of presentations with AOM [58]. This suggests that strategies for guideline implementation and barriers need further exploration.

There are several barriers to the implementation of watchful waiting in practice. It may be perceived by the clinician that parents will be unwilling to accept delayed prescriptions for their children. However, in an outpatient setting, 86% of parents were willing to accept or probably accept a watchful waiting approach [59]. Furthermore, shared decision making, in which parents and clinicians work together to mutually agree on a treatment approach, has been demonstrated to increase the acceptability of delayed prescription and parent satisfaction [60]. Educational initiatives have also proven effective in increasing a watchful waiting approach in the management of AOM in the paediatric emergency department and reducing antimicrobial use [53]. Despite this, guideline adherence with watchful waiting for AOM remains sub-optimal, and research on its barriers to implementation are required. Research to delineate clinicians’ perspectives on the barriers to delayed prescribing are required. There may be a fear of progression of disease and complications among clinicians, a lack of faith in the availability of close clinical follow up, or time constraints associated with additional parental counselling. Furthermore, there is a dearth of literature evaluating other clinical scenarios in paediatrics where delayed prescription may be of benefit.

## 5. Do Structured Culture Follow-Up Programmes in Patients Discharged from the ED with Cultures Pending Improve Antibiotic Prescribing?

ESCMID guidelines for antimicrobial stewardship in emergency departments concluded that the literature in adult populations supported pharmacist-led laboratory follow-up programmes as an important stewardship resource in the ED. They noted that up to 40% of emergency department encounters require post-discharge antimicrobial stewardship intervention, and in adult populations, this intervention reduces the time to positive culture review and primary physician notification, and decreases need for readmission [5,61].

The literature specifically addressing pharmacist-led culture follow up in the paediatric ED is not as robust; however, several studies report the benefits of structured culture follow-up programmes. For example, a nurse-and-clinician-led urine culture follow-up programme in PED patients discharged with presumed UTI resulted in an antibiotic discontinuation rate of up to 84% without any increase in re-attendance for UTI, significantly reducing antibiotic days [62]. Echoing the adult literature, a pharmacist-led culture review programme for urinary and genital culture samples reduced the time to notification of families for thePED, although it did not evaluate the impact of this intervention on return visits or patient outcome [63]. Another study in an emergency department that included 20% paediatric patients demonstrated that involving pharmacists with advanced practice credentials could streamline a culture response programme in the ED, decreasing the median time to perform an intervention with a higher rate of intervention for positive cultures [64]. More generally, embedding antimicrobial stewardship pharmacists within the PEDreduces inappropriate antimicrobial prescribing and may reduce return visits and hospitalisations [65]. Certainly, we can conclude that a pharmacist-led antimicrobial stewardship service has significant potential benefits in the paediatric emergency department, and continued research on the impact of these interventions is desirable.

## 6. Conclusions

The ESCMID guidelines on antimicrobial stewardship in adult emergency departments highlighted four specific evidence-based interventions that are recommended. Here, we aimed to summarise the evidence in paediatric populations for these four domains.

- We found that biomarkers, in particular PCT, may have a role in the risk stratification of paediatric patients in EDs with UTI and in the febrile neonate, but do not replace clinical judgement, particularly for CAP.
- The majority of well-appearing immunocompetent children will gain little benefit and potential harm from the routine use of a blood culture in their work up.
- Evidence supports watchful waiting for otitis media, but implementation appears to be challenging.
- Structured culture follow-up programmes and antimicrobial pharmacist involvement in paediatric emergency department is beneficial, but evidence supporting the impact and sustainability of these programmes are limited.

Overall, the evidence underpinning effective stewardship in this setting remains limited and the certainty of the evidence is low. High-quality implementation studies specifically targeting PED are lacking, and there is an absence of evidence on long-term sustainability, cost effectiveness, and patient-centred outcomes. Further research must prioritise rigorous evaluation of stewardship interventions tailored to the operational realities of the paediatric emergency department to inform evidence-based practice for the next generation.

Antimicrobial stewardship in the PED presents a unique confluence of challenges and opportunities. There are many barriers to optimal antimicrobial prescribing, including limited time and resources, diagnostic uncertainty, high patient turnover, and parental expectations. These contextual pressures can lead to deviations from evidence-based prescribing practices and difficulties in the implementation of antimicrobial stewardship interventions. Many of the interventions we have discussed require robust follow-up programmes to ensure safe care while reducing unnecessary antimicrobial exposure, which may be challenging in health systems where continuity of care is not guaranteed.

Despite these obstacles, a significant proportion of antibiotic courses prescribed in paediatric hospitals commence in the ED, whether the patient is discharged or admitted. They provide a crucial touch point for public education and set an example for appropriate antimicrobial use given the high patient volume and broad reach. The ED is the gatekeeper of the hospital and serves as a strategic frontier for advancing antimicrobial stewardship at an institutional level. Despite the inherent challenges, we believe that investing time and resources to support exemplary antimicrobial use in the paediatric ED will have meaningful benefits that extend far beyond the emergency department.

## Data Availability

No new data were created or analysed in this study. Data sharing is not applicable to this article.

## References

[1] Dyar O.J., Huttner B., Schouten J., Pulcini C. (2017). What is antimicrobial stewardship?. Clin. Microbiol. Infect..

[2] May L., Martín Quirós A., Ten Oever J., Hoogerwerf J., Schoffelen T., Schouten J. (2021). Antimicrobial stewardship in the emergency department: Characteristics and evidence for effectiveness of interventions. Clin. Microbiol. Infect..

[3] Rafferty A., Talendo A.F., Drew R., Fitzpatrick P., Tedford K., Barrett M., Mahomed H., O’Regan S., Delany L., O’Connor S. (2024). Where to start? The Irish Emergency Department Antimicrobial Discharge (EDAD) study: A multicentre, prospective cohort analysis. JAC Antimicrob. Resist..

[4] van de Maat J., van de Voort E., Mintegi S., Gervaix A., Nieboer D., Moll H., Oostenbrink R., European Pediatric Emergency Medicine Study Group (2019). Antibiotic prescription for febrile children in European emergency departments: A cross-secitonal, observational study. Lancet Infect. Dis..

[5] Schoffelen T., Papan C., Carrara E., Eljaaly K., Paul M., Keuleyan E., Quirós A.M., Peiffer-Smadja N., Palos C., May L. (2024). European society of clinical microbiology and infectious diseases guidelines for antimicrobial stewardship in emergency departments (endorsed by European association of hospital pharmacists). Clin. Microbiol. Infect..

[6] Covino M., Buonsenso D., Gatto A., Morello R., Curatole A., Simeoni B., Franceschi F., Chiaretti A. (2022). Determinants of antibiotic prescriptions in a large cohort of children discharged from a pediatric emergency department. Eur. J. Pediatr..

[7] van de Maat J.S., Peeters D., Nieboer D., van Wermeskerken A.M., Smit F.J., Noordzij J.G., Tramper-Stranders G., Driessen G.J.A., Obihara C.C., Punt J. (2020). Evaluation of a clinical decision rule to guide antibiotic prescription in children with suspected lower respiratory tract infection in The Netherlands: A stepped-wedge cluster randomised trial. PLoS Med..

[8] Aabenhus R., Jensen J.U., Jørgensen K.J., Hróbjartsson A., Bjerrum L. (2014). Biomarkers as point-of-care tests to guide prescription of antibiotics in patients with acute respiratory infections in primary care. Cochrane Database Syst. Rev..

[9] Maruna P., Nedelníková K., Gürlich R. (2000). Physiology and genetics of procalcitonin. Physiol. Res..

[10] Kim J.H. (2022). Clinical Utility of Procalcitonin on Antibiotic Stewardship: A Narrative Review. Infect. Chemother..

[11] Linscheid P., Seboek D., Nylen E.S., Langer I., Schlatter M., Becker K.L., Keller U., MülLer B. (2003). In vitro and in vivo calcitonin I gene expression in parenchymal cells: A novel product of human adipose tissue. Endocrinology.

[12] Dark P., Hossain A., McAuley D.F., Brealey D., Carlson G., Clayton J.C., Felton T.W., Ghuman B.K., Gordon A.C., Hellyer T.P. (2024). Biomarker-Guided Antibiotic Duration for Hospitalized Patients with Suspected Sepsis: The ADAPT-Sepsis Randomized Clinical Trial. JAMA.

[13] Schuetz P., Wirz Y., Sager R., Christ-Crain M., Stolz D., Tamm M., Bouadma L., E Luyt C., Wolff M., Chastre J. (2017). Procalcitonin to initiate or discontinue antibiotics in acute respiratory tract infections. Cochrane Database Syst. Rev..

[14] Gerber J.S., Kronman M.P., Ross R.K., Hersh A.L., Newland J.G., Metjian T.A., Zaoutis T.E. (2013). Identifying Targets for Antimicrobial Stewardship in Children’s Hospitals. Infect. Control Hosp. Epidemiol..

[15] Singh C., Verma S., Reddy P., Diamond M.S., Curiel D.T., Patel C., Jain M.K., Redkar S.V., Bhate A.S., Gundappa V. (2023). Phase III Pivotal comparative clinical trial of intranasal (iNCOVACC) and intramuscular COVID 19 vaccine (Covaxin^®^). Npj Vaccines.

[16] Ratageri V.H., Panigatti P., Mukherjee A., Das R.R., Goyal J.P., Bhat J.I., Vyas B., Lodha R., Singhal D., Kumar P. (2022). Role of procalcitonin in diagnosis of community acquired pneumonia in Children. BMC Pediatr..

[17] Stockmann C., Ampofo K., Killpack J., Williams D.J., Edwards K.M., Grijalva C.G., Arnold S.R., A McCullers J., Anderson E.J., Wunderink R.G. (2018). Procalcitonin Accurately Identifies Hospitalized Children with Low Risk of Bacterial Community-Acquired Pneumonia. J. Pediatr. Infect. Dis. Soc..

[18] Roukens A.H.E., Pothast C.R., König M., Huisman W., Dalebout T., Tak T., Azimi S., Kruize Y., Hagedoorn R.S., Zlei M. (2022). Prolonged activation of nasal immune cell populations and development of tissue-resident SARS-CoV-2-specific CD8^+^ T cell responses following COVID-19. Nat. Immunol..

[19] Esposito S., Stefanelli P., Fry N.K., Fedele G., He Q., Paterson P., Tan T., Knuf M., Rodrigo C., Olivier C.W. (2019). Pertussis Prevention: Reasons for Resurgence, and Differences in the Current Acellular Pertussis Vaccines. Front. Immunol..

[20] Mattoo T.K., Spencer J.D. (2024). Biomarkers for urinary tract infection: Present and future perspectives. Pediatr. Nephrol..

[21] Chen S.M., Chang H.M., Hung T.W., Chao Y.H., Tsai J.D., Lue K.H., Sheu J.-N. (2013). Diagnostic performance of procalcitonin for hospitalised children with acute pyelonephritis presenting to the paediatric emergency department. Emerg. Med. J..

[22] Kotoula A., Gardikis S., Tsalkidis A., Mantadakis E., Zissimopoulos A., Deftereos S., Tripsianis G., Manolas K., Chatzimichael A., Vaos G. (2009). Comparative Efficacies of Procalcitonin and Conventional Inflammatory Markers for Prediction of Renal Parenchymal Inflammation in Pediatric First Urinary Tract Infection. Urology.

[23] Bressan S., Andreola B., Zucchetta P., Montini G., Burei M., Perilongo G., Da Dalt L. (2009). Procalcitonin as a predictor of renal scarring in infants and young children. Pediatr. Nephrol. Berl. Ger..

[24] Leroy S., Fernandez-Lopez A., Nikfar R., Romanello C., Bouissou F., Gervaix A., Gurgoze M.K., Bressan S., Smolkin V., Tuerlinckx D. (2013). Association of Procalcitonin with Acute Pyelonephritis and Renal Scars in Pediatric UTI. Pediatrics.

[25] Dagan R., Powell K.R., Hall C.B., Menegus M.A. (1985). Identification of infants unlikely to have serious bacterial infection although hospitalized for suspected sepsis. J. Pediatr..

[26] Baker M.D., Bell L.M., Avner J.R. (1993). Outpatient Management without Antibiotics of Fever in Selected Infants. N. Engl. J. Med..

[27] Hui C., Neto G., Tsertsvadze A., Yazdi F., Tricco A.C., Tsouros S., Skidmore B., Daniel R. (2012). Diagnosis and management of febrile infants (0–3 months). Evid. Rep. Technol. Assess..

[28] Meehan W.P., Fleegler E., Bachur R.G. (2010). Adherence to guidelines for managing the well-appearing febrile infant: Assessment using a case-based, interactive survey. Pediatr. Emerg. Care.

[29] Park J.S., Byun Y.H., Lee J.Y., Lee J.S., Ryu J.M., Choi S.J. (2021). Clinical utility of procalcitonin in febrile infants younger than 3 months of age visiting a pediatric emergency room: A retrospective single-center study. BMC Pediatr..

[30] Milcent K., Faesch S., Gras-Le Guen C., Dubos F., Poulalhon C., Badier I., Marc E., Laguille C., de Pontual L., Mosca A. (2016). Use of Procalcitonin Assays to Predict Serious Bacterial Infection in Young Febrile Infants. JAMA Pediatr..

[31] Christensen D., Polacek C., Sheward D.J., Hanke L., Moliner-Morro A., McInerney G., Murrell B., Hartmann K.T., Jensen H.E., Jungersen G. (2022). Protection against SARS-CoV-2 transmission by a parenteral prime—Intranasal boost vaccine strategy. eBioMedicine.

[32] Maniaci V., Dauber A., Weiss S., Nylen E., Becker K.L., Bachur R. (2008). Procalcitonin in young febrile infants for the detection of serious bacterial infections. Pediatrics.

[33] Rotrosen E., Kupper T.S. (2023). Assessing the generation of tissue resident memory T cells by vaccines. Nat. Rev. Immunol..

[34] Patel D.K., Mohapatra M.K., Thomas A.G., Patel S., Purohit P. (2014). Procalcitonin as a biomarker of bacterial infection in sickle cell vaso-occlusive crisis. Mediterr. J. Hematol. Infect. Dis..

[35] Maziashvili G., Hatabah D., Korman R., Brown L.A., Harris F., Rees C.A., Dampier C., Morris C.R. (2024). Procalcitonin Elevation in Children with Sickle Cell Disease (SCD) Requiring Hospitalization for Acute Vaso-Occlusive Pain (VOE). Blood.

[36] Elenga N., Placide L., Cuadro-Alvarez E., Long L., Njuieyon F., Martin E., Kom-Tchameni R., Defo A., Razafindrakoto S.H., Mrsic Y. (2019). Does Procalcitonin Predict Bacterial Infection in Febrile Children with Sickle Cell Disease?. Indian J. Pediatr..

[37] Damman J., Arias P., Kerner J., Zhang K.Y., Dehghan M., Krishnan G., Nespor C., Bensen R., Park K. (2019). Procalcitonin as a Predictive Marker for Bacteremia in Children with a Central Line and Fever. Hosp. Pediatr..

[38] Kasem A.J., Bulloch B., Henry M., Shah K., Dalton H. (2012). Procalcitonin as a marker of bacteremia in children with fever and a central venous catheter presenting to the emergency department. Pediatr. Emerg. Care.

[39] Andrade A., Bang H., Reddick K., Villaseñor B., Tran N.K., May L. (2023). Evaluation of pharmacist guided intervention using procalcitonin and respiratory virus testing. Am. J. Emerg. Med..

[40] Lacroix L., Papis S., Mardegan C., Luterbacher F., L’Huillier A., Sahyoun C., Keitel K., Mastboim N., Etshtein L., Shani L. (2023). Host biomarkers and combinatorial scores for the detection of serious and invasive bacterial infection in pediatric patients with fever without source. PLoS ONE.

[41] Tan C.D., Van Den Broek B., Womersley R.S., Kaforou M., Hagedoorn N.N., Van Der Flier M., Jackson H., Moll H.A., Snijder R., de Jonge M.I. (2023). A Novel Combination of Host Protein Biomarkers to Distinguish Bacterial from Viral Infections in Febrile Children in Emergency Care. Pediatr. Infect. Dis. J..

[42] Jullien S., Richard-Greenblatt M., Ngai M., Lhadon T., Sharma R., Dema K., Kain K.C., Bassat Q. (2022). Performance of host-response biomarkers to risk-stratify children with pneumonia in Bhutan. J. Infect..

[43] Fernandes C.D., Arriaga M.B., Costa M.C.M., Costa M.C.M., Costa M.H.M., Vinhaes C.L., Silveira-Mattos P.S., Fukutani K.F., Andrade B.B. (2019). Host Inflammatory Biomarkers of Disease Severity in Pediatric Community-Acquired Pneumonia: A Systematic Review and Meta-analysis. Open Forum Infect. Dis..

[44] Habgood-Coote D., Wilson C., Shimizu C., Barendregt A.M., Philipsen R., Galassini R., Calle I.R., Workman L., Agyeman P.K., Ferwerda G. (2023). Diagnosis of childhood febrile illness using a multi-class blood RNA molecular signature. Med.

[45] Peng K., Zhang X., Li Q., Luo Z. (2025). Presepsin as a diagnostic biomarker for sepsis across neonates, children, and adults: A meta-analysis. Biomol. Biomed..

[46] Nusman C., Schouten S.M., Nagelkerke S., Keuning M.W., Borensztajn D., Plötz F.B., Visser D.H., Hol J. (2026). The Role of Presepsin in Differentiating Between Bacterial and Viral Infections in Young Infants With Fever of Unknown Origin on the Emergency Department. Pediatr. Infect. Dis. J..

[47] Bradley J.S., Byington C.L., Shah S.S., Alverson B., Carter E.R., Harrison C., Kaplan S.L., Mace S.E., McCracken G.H., Moore M.R. (2011). The Management of Community-Acquired Pneumonia in Infants and Children Older Than 3 Months of Age: Clinical Practice Guidelines by the Pediatric Infectious Diseases Society and the Infectious Diseases Society of America. Clin. Infect. Dis..

[48] Stevens D.L., Bisno A.L., Chambers H.F., Dellinger E.P., Goldstein E.J.C., Gorbach S.L., Hirschmann J.V., Kaplan S.L., Montoya J.G., Wade J.C. (2014). Practice Guidelines for the Diagnosis and Management of Skin and Soft Tissue Infections: 2014 Update by the Infectious Diseases Society of America. Clin. Infect. Dis..

[49] Bryant P.A., Bitsori M., Vardaki K., Vaezipour N., Khan M., Buettcher M. (2025). Guidelines for Complicated Urinary Tract Infections in Children: A Review by the European Society for Pediatric Infectious Diseases. Pediatr. Infect. Dis. J..

[50] Bachur R., Caputo G.L. (1995). Bacteremia and meningitis among infants with urinary tract infections. Pediatr. Emerg. Care.

[51] Hoberman A., Wald E.R., Hickey R.W., Baskin M., Charron M., Majd M., Kearney D.H., Reynolds E.A., Ruley J., Janosky J.E. (1999). Oral versus initial intravenous therapy for urinary tract infections in young febrile children. Pediatrics.

[52] Honkinen O., Jahnukainen T., Mertsola J., Eskola J., Ruuskanen O. (2000). Bacteremic urinary tract infection in children. Pediatr. Infect. Dis. J..

[53] Sun D., McCarthy T.J., Liberman D.B. (2017). Cost-Effectiveness of Watchful Waiting in Acute Otitis Media. Pediatrics.

[54] Spurling G.K.P., Del Mar C.B., Dooley L., Foxlee R., Farley R. (2013). Delayed antibiotics for respiratory infections. Cochrane Database Syst. Rev..

[55] Mas-Dalmau G., Villanueva López C., Gorrotxategi Gorrotxategi P., Argüelles Prendes E., Espinazo Ramos O., Valls Duran T., Alonso M.E.G., Viana M.P.C., Bada T.M., Fernández M.E.V. (2021). Delayed Antibiotic Prescription for Children with Respiratory Infections: A Randomized Trial. Pediatrics.

[56] NICE Cough (Acute): Antimicrobial Prescribing. https://www.nice.org.uk/guidance/ng120/resources/cough-acute-antimicrobial-prescribing-pdf-66141652166341.

[57] NICE Otitis Media (Acute): Antimicrobial Prescribing. https://www.nice.org.uk/guidance/ng91.

[58] Spoială E.L., Stârcea I.M., Ioniuc I.K., Cozma R.S., Rusu D.C., Bozomitu L., Lupu V.V., Haliţchi C.O.I., Roşu V.E., Roşu S.T. (2023). Watchful Waiting in Pediatric Acute Otitis Media: A Real Practice Approach or an Intangible Desideratum?. Medicina.

[59] Broides A., Bereza O., Lavi-Givon N., Fruchtman Y., Gazala E., Leibovitz E. (2016). Parental acceptability of the watchful waiting approach in pediatric acute otitis media. World J. Clin. Pediatr..

[60] Merenstein D., Diener-West M., Krist A., Pinneger M., Cooper L.A. (2005). An assessment of the shared-decision model in parents of children with acute otitis media. Pediatrics.

[61] Boot A., Weideling A., Wilson A., Burnham D., Ward S.P. (2024). Impact of a pharmacy-driven culture callback protocol on antimicrobial therapy optimization in the emergency department. J. Am. Pharm. Assoc..

[62] Saha D., Patel J., Buckingham D., Thornton D., Barber T., Watson J.R. (2017). Urine Culture Follow-up and Antimicrobial Stewardship in a Pediatric Urgent Care Network. Pediatrics.

[63] Tweedle J., Mercado E., Truesdale N., Leonard D., Nesiama J.A.O. (2020). Decreasing review and notification times of genital and urine cultures in a pediatric emergency department: An observational before and after study. J. Am. Coll. Emerg. Physicians Open.

[64] Cornell W.K., Hile G., Stone T., Hannum J., Reichert M., Hollinger M.K. (2022). Impact of advanced practice pharmacists on a culture response program in the emergency department. Am. J. Health-Syst. Pharm..

[65] MacMillan K.M., MacInnis M., Fitzpatrick E., Hurley K.F., MacPhee S., Matheson K., Black E.K. (2019). Evaluation of a pharmacist-led antimicrobial stewardship service in a pediatric emergency department. Int. J. Clin. Pharm..

